# Clinical Profile, Precipitating Factors, and Predictors of In-Hospital Mortality in Patients With Acute-on-Chronic Liver Failure: A Cross-Sectional Study From a Tertiary Care Centre in Central India

**DOI:** 10.7759/cureus.117522

**Published:** 2026-10-06

**Authors:** Eeshan Aradhye, Tanuja Manohar, Fahad Idrees Shaikh

**Affiliations:** 1 General Medicine, NKP Salve Institute of Medical Sciences and Research Centre and Lata Mangeshkar Hospital, Nagpur, IND; 2 Medicine, NKP Salve Institute of Medical Sciences and Research Centre and Lata Mangeshkar Hospital, Nagpur, IND

**Keywords:** aarc score, acute-on-chronic liver failure, apasl, cirrhosis, india, in-hospital mortality, meld score, sofa score

## Abstract

Background

Acute-on-chronic liver failure (ACLF) is a distinct clinical syndrome characterised by acute hepatic decompensation in patients with chronic liver disease (CLD), frequently accompanied by extrahepatic organ failure and high short-term mortality. Region-specific data on the clinical profile, precipitating factors, and factors associated with outcome in ACLF remain limited in Indian tertiary care settings.

Objectives

To describe the clinical features, biochemical parameters, precipitating factors, organ dysfunction, and severity-score profile of patients with ACLF, and to evaluate factors associated with in-hospital mortality, using the European Association for the Study of the Liver-Chronic Liver Failure (EASL-CLIF) and Asian Pacific Association for the Study of the Liver (APASL) ACLF Research Consortium (AARC) scoring systems.

Methods

This prospective observational cohort study enrolled 75 consecutive patients diagnosed with ACLF as per APASL criteria at a tertiary care teaching hospital. Demographic, clinical, biochemical, and severity-score data [Child-Turcotte-Pugh (CTP), model for end-stage liver disease (MELD), MELD-Na, sequential organ failure assessment (SOFA), and AARC scores] were recorded at admission, and patients were followed prospectively until in-hospital discharge or death. Categorical variables were compared using the chi-square test (with Yates’ continuity correction for 2×2 tables) and continuous variables using the independent-samples t-test; all analyses were univariate.

Results

Of 75 patients, 66 (88.0%) were male, with a mean age of 47.0 ± 13.7 years. Jaundice (72/75, 96.0%) and ascites (68/75, 90.7%) were the commonest presenting features. Alcohol-related CLD (41/75, 54.7%) was the leading aetiology, and infection (25/75, 33.3%) - predominantly spontaneous bacterial peritonitis - was the commonest precipitating event. Fifty patients (50/75, 66.7%) were CTP class C, 39/75 (52.0%) had a MELD score ≥30, and 34/75 (45.3%) had an AARC score ≥10. In-hospital mortality was 46.7% (35/75) and rose significantly with ACLF grade (14.3% [4/28], 51.9% [14/27], and 85.0% [17/20] in grades 1, 2, and 3, respectively; χ²=23.90, p<0.001). Non-survivors had significantly higher bilirubin, creatinine, international normalised ratio (INR), MELD, MELD-Na, SOFA, AARC score, and serum lactate, and significantly lower serum sodium than survivors (all p<0.01). Multi-organ failure (26/35, 74.3% vs 4/40, 10.0%, p<0.0001) and an AARC score ≥10 (27/35, 77.1% vs 7/40, 17.5%, p<0.0001) showed the strongest univariate association with mortality.

Conclusion

ACLF in this Indian cohort predominantly affected middle-aged men with alcohol-related CLD, commonly precipitated by infection. In-hospital mortality was high and closely tracked the burden of organ failure. Hyperbilirubinaemia, coagulopathy, renal dysfunction, hyponatraemia, hyperlactataemia, and elevated MELD, SOFA, and AARC scores were factors significantly associated with in-hospital death on univariate analysis, supporting further evaluation of organ failure-based scoring systems for risk stratification in ACLF.

## Introduction

Chronic liver disease (CLD) is a major and growing public health burden worldwide, particularly in low- and middle-income countries. Patients with established CLD may remain clinically compensated for prolonged periods; however, an acute insult can precipitate sudden and severe deterioration in hepatic function, producing a distinct clinical syndrome termed acute-on-chronic liver failure (ACLF). ACLF is characterised by rapid progression, the development of single or multiple organ failures, and short-term mortality far exceeding that seen in stable cirrhosis or simple acute decompensation [1,2].

ACLF is now recognised as a syndrome distinct from both acute liver failure and decompensated cirrhosis: unlike acute liver failure, it occurs on a background of pre-existing CLD (previously diagnosed or occult), and unlike simple decompensation, it is marked by an exaggerated systemic inflammatory response, rapid extrahepatic organ dysfunction, and a potentially reversible course if identified and managed early [2,3]. A persistent challenge has been the heterogeneity of diagnostic criteria. The Asian Pacific Association for the Study of the Liver (APASL) defines ACLF primarily as a liver-centred disorder occurring in patients with or without cirrhosis, whereas the European Association for the Study of the Liver-Chronic Liver Failure (EASL-CLIF) Consortium's CLIF Acute-oN-ChrONic liver failure In Cirrhosis (CANONIC) definition is restricted to patients with cirrhosis and incorporates extrahepatic organ dysfunction across six organ systems [2,4,5]. The American Association for the Study of Liver Diseases (AASLD) and, more recently, the multisociety 'Kyoto Consensus' have attempted to reconcile these frameworks, but no single definition has achieved universal acceptance, which continues to affect comparability of prevalence, phenotype, and outcome data across regions [5-8].

The pathophysiology of ACLF reflects a complex interplay of hepatic injury, systemic inflammation, immune dysfunction, and circulatory derangement, in which the severity and number of organ failures - rather than the degree of hepatic dysfunction alone - determine short-term prognosis [1,3]. The APASL ACLF Research Consortium (AARC) subsequently derived a dynamic, organ failure-weighted score (the AARC score) from a large multinational Asian cohort, incorporating total bilirubin, creatinine, serum lactate, international normalised ratio (INR), and hepatic encephalopathy, which outperformed the model for end-stage liver disease (MELD) and chronic liver failure-sequential organ failure assessment (CLIF-SOFA) in predicting mortality [6].

The aetiological and precipitating-factor spectrum of ACLF also shows marked regional variation. Alcohol-related liver disease, viral hepatitis (particularly hepatitis B and E), and bacterial infections - especially spontaneous bacterial peritonitis - are the dominant hepatic and non-hepatic triggers reported from Indian cohorts, in contrast to alcoholic hepatitis and gastrointestinal bleeding, which predominate in Western series [9-11]. Traditional liver-specific scores such as Child-Turcotte-Pugh (CTP) and MELD, while useful in stable cirrhosis, have limited discriminatory value in ACLF; organ failure-based systems such as the Sequential Organ Failure Assessment (SOFA), CLIF-C ACLF, and AARC scores have shown superior prognostic performance in prior validation cohorts and now guide decisions on intensive care admission and referral for transplantation [12-14].

Despite these advances, in-hospital and short-term mortality in ACLF remains alarmingly high, particularly in resource-limited settings, with several Indian cohorts reporting 28-day to in-hospital mortality of 30-50% or more, rising sharply with the number of organ failures [11,15-17]. Delayed presentation, limited access to specialised hepatology and critical care services, and heterogeneous application of diagnostic criteria further compound outcomes in the Indian context. Comprehensive, institution-level data correlating clinical presentation, precipitating factors, organ dysfunction, and validated severity scores with in-hospital outcomes remain limited from Indian tertiary centres [18,19].

Given the dynamic and potentially reversible nature of ACLF, early recognition and detailed characterisation of the local clinical profile are essential for developing context-appropriate management strategies and for optimising triage and resource allocation.

This study aimed to: (i) describe the clinical characteristics, precipitating factors, laboratory derangements, organ dysfunction, and severity-score profile among patients with ACLF admitted to a tertiary care centre (descriptive objective); and (ii) evaluate factors associated with in-hospital mortality in this cohort, using the APASL diagnostic framework and the AARC and organ failure-based scoring systems (prognostic objective). Consistent with the univariate statistical methods used (described below), associations identified in this study are reported as factors associated with mortality rather than as independent predictors, which would require multivariable analysis.

## Materials and methods

Study design and setting

This was a prospective observational cohort study with both descriptive and analytical components, conducted in the Department of General Medicine, NKP Salve Institute of Medical Sciences and Research Center and Lata Mangeshkar Hospital, Nagpur, Maharashtra, India, from August 15, 2024, to August 15, 2026. All consecutive patients admitted with acute hepatic decompensation on a background of chronic liver disease (CLD) were screened for eligibility. Patients were enrolled at the time of admission and followed prospectively, with repeated clinical and biochemical assessment, until in-hospital discharge or death; the study design is therefore a prospective observational cohort study rather than a cross-sectional study, reflecting this within-admission longitudinal follow-up of each patient.

Sample size calculation

The sample size was estimated using the standard formula for estimating a population proportion, \[ n = \frac{Z_{\alpha/2}^{2}\,P(1-P)}{E^{2}}, \] where $Z_{\alpha/2}=1.96$ (95\% confidence interval), *P=5%* (estimated prevalence of ACLF among hospitalised CLD patients, adopted as a conservative estimate based on institutional admission patterns and previously published regional data, in the absence of a precise local prevalence figure), and *E=5%* (absolute precision).

This yielded a minimum calculated sample size of 72.99, rounded up to 75; accordingly, 75 consecutive patients fulfilling the inclusion criteria were enrolled using a consecutive sampling method. Complete baseline, in-hospital, and outcome data were available for all 75 enrolled patients for the variables reported in this manuscript; no values required imputation.

Participants

Inclusion Criteria

Patients aged more than 18 years presenting with an acute hepatic insult, with or without prior hepatic decompensation, diagnosed as ACLF as per APASL criteria [4,5], who provided written informed consent.

Exclusion Criteria

Patients presenting initially with gastrointestinal haemorrhage, and patients presenting with septicaemia at onset unless the primary source of infection was hepatic.

ACLF was diagnosed according to the APASL definition as an acute hepatic insult manifesting as jaundice (serum bilirubin ≥5 mg/dL) and coagulopathy (INR ≥1.5 or prothrombin activity <40%), complicated within four weeks by ascites and/or hepatic encephalopathy, in a patient with previously diagnosed or undiagnosed chronic liver disease [4,5]. Cirrhosis was diagnosed on a combination of clinical features, biochemical parameters, imaging findings, or prior liver biopsy showing F4 fibrosis. Acute hepatic insults were defined as viral superinfection (hepatitis A or E virus), viral reactivation (hepatitis B virus), continued alcohol consumption, autoimmune flare, or drug-induced liver injury (including antitubercular and antiepileptic drugs). Non-hepatic (extrahepatic) insults were classified as variceal bleeding or sepsis [including spontaneous bacterial peritonitis (SBP), urinary tract infection, respiratory tract infection, cellulitis, and spontaneous bacteraemia]. For this study, the following operational definitions were applied to the two commonest precipitating-event categories: infection as a precipitating event was defined as a new or clinically evolving infection identified within 48 hours of presentation and judged by the treating team to be the primary trigger for acute decompensation, supported by clinical, laboratory, and radiological evidence (e.g. an ascitic fluid absolute neutrophil count ≥250 cells/mm³ for SBP, a positive urine culture with symptoms for urinary tract infection, or a new radiographic infiltrate with clinical correlation for pneumonia); binge alcohol consumption was defined as self-reported ingestion of more than 80-100 g of alcohol per day for at least four weeks, or a clear acute increase in intake above the patient's own baseline pattern, in the period immediately preceding presentation. Silent CLD was defined as previously undiagnosed CLD with no prior decompensation, and overt CLD as a prior diagnosis of cirrhosis with or without previous decompensation.

Data collection

Data were collected on a pre-structured proforma comprising demographic details, clinical features, laboratory investigations, severity scores, and outcome parameters. At admission, the following baseline variables were recorded for all patients: demographic and clinical details (age, sex, presenting symptoms, and clinical findings); laboratory parameters (haemogram, liver function tests, INR, renal function tests, and serum electrolytes); severity and prognostic scores (CTP, MELD, MELD-Na, SOFA, and CLIF-SOFA/AARC scores) at baseline, repeated at weekly intervals during hospitalisation; and the aetiology of underlying CLD, precipitating cause of acute decompensation, and in-hospital outcome. A detailed history focused specifically on alcohol consumption, drug intake, haematemesis, melaena, and previously diagnosed viral hepatitis. All patients were screened for hepatitis B surface antigen, IgM hepatitis B core antibody, IgM anti-hepatitis A virus antibody, and IgM anti-hepatitis E virus antibody. Variables in this manuscript are presented according to the time of ascertainment: baseline characteristics and severity scores were assessed at the time of admission, whereas complications and supportive-care interventions (sepsis, hepatorenal syndrome, invasive ventilation, and renal replacement therapy) that developed or were instituted during the course of hospitalisation are reported separately; these are therefore in-hospital events rather than baseline variables, and may represent consequences of disease progression rather than baseline risk factors (see Limitations).

Procedure

All patients received standard-of-care therapy as per institutional protocol. Patients with hepatitis B infection were initiated on tenofovir or entecavir. Empirical broad-spectrum antibiotics were initiated for suspected infection pending culture results and de-escalated according to sensitivity reports; nutritional support and correction of electrolyte and coagulation abnormalities were provided per institutional protocol, and renal replacement therapy was provided when indicated. Patients with variceal bleeding underwent endoscopic variceal ligation. SBP and hepatorenal syndrome were managed according to standard recommendations, and lactulose with rifaximin was administered for hepatic encephalopathy. The need for ventilatory support and choice of antibiotics were determined by the treating physicians. No patient underwent liver transplantation during the study period. Patients were followed throughout their hospital stay until discharge or death.

Outcome measures

The primary outcome was in-hospital mortality. Severity of liver disease was assessed using the CTP, MELD, and modified SOFA scores. The MELD score was calculated using the standard formula: MELD=3.78×ln[serum bilirubin (mg/dL)] + 11.2×ln[INR] + 9.57×ln[serum creatinine (mg/dL)] + 6.43. Systemic inflammatory response syndrome (SIRS) was defined by the presence of ≥ 2 of: fever (>38.3°C) or hypothermia (<35.6°C, rectal); tachycardia (>90/min); tachypnoea (>20/min or PaCO₂ <32 mmHg, or need for mechanical ventilation); and an abnormal leukocyte count (>12,000/mm³, <4,000/mm³, or >10% immature forms). In patients with alcohol-associated liver disease, Maddrey's discriminant function was calculated as 4.6×[prothrombin time (seconds) − control] + serum bilirubin (mg/dL). Organ failure was defined as a SOFA sub-score ≥3 for the relevant organ system, and multi-organ failure as ≥2 extrahepatic organ failures. ACLF grade was assigned according to the number of extrahepatic organ failures present at any point during the course of illness, per APASL criteria: grade 1, single organ failure; grade 2, two organ failures; and grade 3, three or more organ failures. This ACLF grade is conceptually related to, but numerically distinct from, the AARC score category: the APASL-AARC score (range 5-15, incorporating total bilirubin, creatinine, INR, serum lactate, and hepatic encephalopathy grade) was used separately to categorise severity into three bands (5-7, 8-10, and 11-15) [4-6]. Because ACLF grade (based on organ-failure count) and AARC score category (a continuous composite score) classify overlapping but non-identical dimensions of disease severity, the group sizes differ between the two classification systems; both are reported, as each provides complementary prognostic information. All severity scores were calculated at baseline and repeated at weekly intervals.

Statistical analysis

Data were analysed using SPSS, version 24 (IBM Corporation, Armonk, USA). Continuous variables are expressed as mean ± standard deviation (SD) and were compared between groups (survivors vs non-survivors) using the independent-samples (unpaired) Student's t-test; continuous variables were assessed for approximate normality by visual inspection of their distribution before the use of this parametric test, and the t-test was considered appropriate given their approximately symmetric distribution and the sample size in each group. Categorical variables are expressed as frequencies and percentages and were compared using the chi-square test; Yates' continuity correction was applied for 2×2 contingency tables, and the Pearson chi-square test (without continuity correction) was used for tables with more than two categories (e.g. mortality across ACLF grades). A two-tailed p-value <0.05 was considered statistically significant. Test statistics accompanying each p-value in the results tables below (t for continuous comparisons, χ² for categorical comparisons) were derived from the reported summary data using these methods, with degrees of freedom noted in each table. All analyses performed in this study were univariate; no multivariable or multivariate regression modelling was undertaken. Accordingly, variables found to be statistically associated with mortality on these univariate comparisons are reported throughout this manuscript as factors associated with in-hospital mortality, and not as independent predictors, since independence from confounding and from one another (including overlap between individual laboratory variables and the composite scores of which they are components) was not formally assessed.

Ethical considerations

The study protocol was approved by the Institutional Ethics Committee at NKP Salve Institute of Medical Sciences & Research Center and Lata Mangeshkar Hospital (IEC Reference No. 45/2024; approval date: August 9, 2024). Written informed consent was obtained from all participants prior to enrolment, and confidentiality of patient data was maintained throughout the study.

## Results

A total of 75 patients meeting APASL criteria for ACLF were enrolled and evaluated during the study period. Their demographic, clinical, biochemical, and outcome profiles are presented below in Table 1. The male-to-female ratio was approximately 7.3:1. The largest proportion of patients belonged to the 41-50-year age group.

**Table 1:** Baseline demographic profile (N=75)

Variable	Category	N (%)
Sex	Male	66 (88.0)
	Female	9 (12.0)
Age group (years)	18–30	7 (9.3)
	31–40	15 (20.0)
	41–50	22 (29.3)
	51–60	18 (24.0)
	>60	13 (17.3)
Age (years), mean ± SD	47.0 ± 13.7	—

Table 2 summarizes the presenting clinical features. Jaundice (72/75, 96.0%) and ascites (68/75, 90.7%) were seen in the overwhelming majority of patients, and half the cohort (38/75, 50.7%) had hepatic encephalopathy at presentation, reflecting advanced hepatic decompensation at the time of admission.

**Table 2:** Clinical presentation at admission (N=75)

Clinical feature	N (%)
Jaundice	72 (96.0)
Ascites/abdominal distension	68 (90.7)
Hepatic encephalopathy	38 (50.7)
Oliguria/decreased urine output	34 (45.3)
Fever	25 (33.3)
Abdominal pain	21 (28.0)
Gastrointestinal bleeding	20 (26.7)
Peripheral oedema	18 (24.0)

Table 3 shows the distribution of aetiologies underlying CLD. Alcohol-related liver disease was the leading cause, accounting for more than half of all cases (41/75, 54.7%), followed at a distance by HBV-related CLD (14/75, 18.7%); a definite aetiology could not be established in 6.7% (5/75) of patients.

**Table 3:** Aetiology of underlying chronic liver disease (N=75) CLD: chronic liver disease; HBV: hepatitis B virus; HCV: hepatitis C virus; NASH: non-alcoholic steatohepatitis; MAFLD: metabolic dysfunction-associated fatty liver disease.

Aetiology	N (%)
Alcohol-related CLD	41 (54.7)
HBV-related CLD	14 (18.7)
NASH/MAFLD	7 (9.3)
HCV-related CLD	4 (5.3)
Autoimmune/other	4 (5.3)
Undetermined	5 (6.7)
Total	75 (100.0)

Table 4 details the acute hepatic insult identified as per APASL criteria. Continued alcohol consumption or superadded alcoholic hepatitis was the commonest identifiable hepatic trigger (27/75, 36.0%), followed equally by hepatitis E virus (HEV) superinfection and hepatitis B virus (HBV) reactivation (12/75, 16.0% each); no definite hepatic insult could be identified in 14.7% (11/75) of patients.

**Table 4:** Acute hepatic insult/precipitating hepatic event (APASL triggers) (N=75) HEV: hepatitis E virus; HBV: hepatitis B virus; HAV: Hepatitis A virus; APASL: Asian Pacific Association for the Study of the Liver.

Acute hepatic insult	N (%)
Alcohol-associated hepatitis/continued alcohol use	27 (36.0)
HEV superinfection	12 (16.0)
HBV reactivation	12 (16.0)
Drug-induced liver injury	6 (8.0)
Autoimmune flare	4 (5.3)
HAV superinfection	3 (4.0)
No identifiable trigger	11 (14.7)

Table 5 classifies the overall precipitating event for the episode of acute decompensation, which need not be confined to a hepatic insult. Infection was the single commonest precipitant (25/75, 33.3%), closely followed by binge alcohol consumption (22/75, 29.3%), together accounting for almost two-thirds of all episodes. Among the 25 patients precipitated by infection, SBP was the commonest focus (12/25, 48.0% of infected patients; 12/75, 16.0% of the total cohort), followed by urinary tract infection (7/25, 28.0%) and pneumonia (6/25, 24.0%).

**Table 5:** Overall precipitating event category and, among patients with infection, the documented infection focus (N=75)

Precipitating event	N (%)
Infection	25 (33.3)
Binge alcohol consumption	22 (29.3)
Viral reactivation	8 (10.7)
Gastrointestinal bleed	7 (9.3)
Drug-induced	6 (8.0)
No definite factor identified	7 (9.3)

Table 6 presents the cohort's baseline laboratory profile. Patients had, on average, marked hyperbilirubinaemia (14.6 ± 7.2 mg/dL, reference 0.3-1.2), a prolonged INR (2.3 ± 0.8, reference 0.8-1.2), moderate anaemia (haemoglobin 9.1 ± 1.9 g/dL), thrombocytopenia (118 ± 62 ×10³/µL, reference 150-450), and impaired renal function (creatinine 1.9 ± 1.2 mg/dL, reference 0.6-1.3) with mild hyponatraemia (sodium 131 ± 6 mEq/L, reference 135-145), reflecting substantial hepatic synthetic dysfunction accompanied by early renal compromise at presentation.

**Table 6:** Baseline laboratory parameters (N=75) *Reference ranges are standard adult laboratory normal values provided for context and may vary slightly between laboratories; SD: standard deviation; M: male; F: female; AST: aspartate aminotransferase; ALT: alanine aminotransferase; INR: international normalized ratio.

Parameter	Mean ± SD	Reference range*
Haemoglobin (g/dL)	9.1 ± 1.9	13.0–17.0 (M); 12.0–15.0 (F)
Total leukocyte count (×10³/µL)	12.8 ± 5.1	4.0–11.0
Platelet count (×10³/µL)	118 ± 62	150–450
Total bilirubin (mg/dL)	14.6 ± 7.2	0.3–1.2
AST (IU/L)	182 ± 110	8–40
ALT (IU/L)	94 ± 62	7–56
Serum albumin (g/dL)	2.6 ± 0.5	3.5–5.0
INR	2.3 ± 0.8	0.8–1.2
Serum creatinine (mg/dL)	1.9 ± 1.2	0.6–1.3
Serum sodium (mEq/L)	131 ± 6	135–145

Table 7 presents these laboratory values as clinically relevant threshold categories. Over half the cohort had an INR exceeding 2.0 (40/75, 53.3%) and thrombocytopenia below 100,000/µL (39/75, 52.0%), while severe hyperbilirubinaemia (>20 mg/dL) and clinically significant hyperlactataemia (≥2 mmol/L) were each present in around 44-48% (36/75 and 33/75, respectively) of patients, indicating a substantial proportion of the cohort had high-grade biochemical derangement at admission.

**Table 7:** Key laboratory derangements at admission (N=75) *Reference ranges are standard adult laboratory normal values provided for context and may vary slightly between laboratories; the thresholds listed in this table are clinically established cut-offs used for risk categorisation rather than the laboratory normal range itself. INR: international normalized ratio.

Parameter	N (%)	Reference range*
Total bilirubin >20 mg/dL	36 (48.0)	0.3–1.2 mg/dL
INR >2.0	40 (53.3)	0.8–1.2
Serum creatinine ≥2.0 mg/dL	26 (34.7)	0.6–1.3 mg/dL
Serum sodium <130 mEq/L	31 (41.3)	135–145 mEq/L
Platelets <100,000/µL	39 (52.0)	150,000–450,000/µL
Serum lactate ≥2 mmol/L	33 (44.0)	0.5–2.0 mmol/L
Serum lactate ≥4 mmol/L	14 (18.7)	0.5–2.0 mmol/L

Table 8 shows how patients were distributed across the standard severity-score categories. Two-thirds of the cohort (50/75, 66.7%) were in CTP class C, and over half had a MELD score ≥30 (39/75, 52.0% combined across the 30-39 and ≥40 bands). By AARC grading, patients were fairly evenly split across the three severity bands, with a slightly higher proportion in the intermediate (8-10, 29/75, 38.7%) and severe (11-15, 28/75, 37.3%) categories than in the mild category (5-7, 18/75, 24.0%), confirming that most patients presented with moderate-to-severe disease.

**Table 8:** Distribution of severity score categories at baseline (N=75) CTP: Child-Turcotte-Pugh; MELD: model for end-stage liver disease; SOFA: sequential organ failure assessment; AARC: ACLF Research Consortium.

Score	Category	N (%)
CTP class	A	3 (4.0)
	B	22 (29.3)
	C	50 (66.7)
MELD score	<20	5 (6.7)
	20–29	31 (41.3)
	30–39	27 (36.0)
	≥40	12 (16.0)
SOFA score	≤5	27 (36.0)
	6–7	16 (21.3)
	8–9	12 (16.0)
	≥10	20 (26.7)
AARC score	5–7	18 (24.0)
	8–10	29 (38.7)
	11–15	28 (37.3)

Table 9 outlines the pattern of individual organ failures (SOFA sub-score ≥3) alongside the prevalence of key severity thresholds. Liver failure (34/75, 45.3%), cerebral failure (30/75, 40.0%), and coagulation failure (29/75, 38.7%) were the most common individual organ failures, while respiratory and circulatory failure were less frequent (14/75, 18.7% and 12/75, 16.0%, respectively). Multi-organ failure, defined as ≥2 extrahepatic organ failures, was present in 40.0% (30/75) of the cohort, and nearly half met SIRS criteria (34/75, 45.3%) at admission.

**Table 9:** Pattern of organ failure (SOFA sub-score ≥3) and prevalence of key severity thresholds at baseline (N=75) CTP: Child-Turcotte-Pugh; MELD: model for end-stage liver disease; SOFA: sequential organ failure assessment; AARC: ACLF Research Consortium; SIRS: systemic inflammatory response syndrome.

Organ system / parameter	N (%)
Liver failure	34 (45.3)
Coagulation failure	29 (38.7)
Cerebral (brain) failure	30 (40.0)
Kidney (renal) failure	23 (30.7)
Respiratory failure	14 (18.7)
Circulatory failure	12 (16.0)
CTP class C	50 (66.7)
MELD ≥30	39 (52.0)
MELD-Na ≥30	41 (54.7)
SOFA ≥8	32 (42.7)
AARC score ≥10	34 (45.3)
SIRS present	34 (45.3)
Multi-organ failure (≥2 extrahepatic organ failures)	30 (40.0)

Table 10 summarizes the in-hospital outcome of the cohort. Of the 75 patients, 40 (53.3%) survived to discharge while 35 (46.7%) died during hospitalisation, an in-hospital mortality rate consistent with the high short-term case-fatality widely reported in ACLF. Invasive ventilatory support was required in 13/75 (17.3%) of patients overall.

**Table 10:** In-hospital outcome (N=75)

Outcome	N (%)
Discharged/survived	40 (53.3)
In-hospital mortality	35 (46.7)
Required invasive ventilatory support	13 (17.3)

Table 11 demonstrates a clear, statistically significant gradient of mortality across ACLF grades, rising from 14.3% (4/28) in grade 1 to 51.9% (14/27) in grade 2 and 85.0% (17/20) in grade 3 (χ²=23.90, df=2, p<0.001), confirming that increasing ACLF grade is strongly and independently associated with a higher risk of in-hospital death.

**Table 11:** Mortality according to ACLF grade (N=75) *Pearson chi-square test across the three ACLF grades (3×2 contingency table). ACLF: acute-on-chronic liver failure.

ACLF grade	Survived (n)	Expired (n)	Mortality (%)	Test statistic	p-value
Grade 1	24	4	14.3	χ²=23.90 (df=2)*	<0.001
Grade 2	13	14	51.9		
Grade 3	3	17	85		
Total	40	35	46.7		

Table 12 compares baseline laboratory parameters and severity scores between survivors and non-survivors. Non-survivors had significantly higher total bilirubin, creatinine, INR, CLIF-SOFA, MELD, MELD-Na, SOFA, and AARC scores, and significantly lower serum sodium than survivors (all p≤0.006), with the largest separation seen for the AARC score (11.4 ± 1.9 vs 8.2 ± 1.6, t=7.92, p<0.0001), indicating that composite organ failure-based scores discriminated outcome more strongly than any single laboratory parameter.

**Table 12:** Laboratory parameters and severity scores by in-hospital outcome (survivors vs non-survivors) *Independent-samples (unpaired) Student's t-test, pooled variance, degrees of freedom=73. SD: standard deviation; INR: international normalized ratio; CLIF-SOFA: chronic liver failure-sequential organ failure assessment; MELD: model for end-stage liver disease; SOFA: sequential organ failure assessment; AARC: ACLF Research Consortium.

Parameter	Survivors (N=40) Mean ± SD	Non-survivors (N=35) Mean ± SD	Test statistic (t, df=73)*	p-value
Total bilirubin (mg/dL)	12.9 ± 6.6	17.2 ± 7.4	t=2.66	0.006
Serum creatinine (mg/dL)	1.4 ± 0.7	2.7 ± 1.4	t=5.18	<0.001
INR	2.0 ± 0.6	2.7 ± 0.9	t=4.01	<0.001
Serum sodium (mEq/L)	133 ± 5	128 ± 6	t=3.94	<0.001
CLIF-SOFA score	7.1 ± 2.3	11.2 ± 2.6	t=7.25	<0.001
MELD score	25.6 ± 5.9	34.2 ± 6.8	t=5.87	<0.0001
MELD-Na score	27.0 ± 6.1	36.1 ± 6.6	t=6.20	<0.0001
SOFA score	6.5 ± 2.3	10.8 ± 2.7	t=7.45	<0.0001
AARC score	8.2 ± 1.6	11.4 ± 1.9	t=7.92	<0.0001

Table 13 shows that neither age nor sex differed significantly between those who died and those who survived (p=0.101 and p=0.334, respectively), indicating that, unlike disease severity, baseline demographic characteristics did not independently influence in-hospital outcome in this cohort.

**Table 13:** Demographic factors by in-hospital outcome *Independent-samples t-test for age; chi-square test with Yates’ continuity correction for sex. SD: standard deviation.

Variable	Died (N=35)	Survived (N=40)	Test statistic*	p-value
Age (years), mean ± SD	49.8 ± 13.2	44.6 ± 13.6	t=1.68 (df=73)	0.101
Male sex, n (%)	32 (91.4)	34 (85.0)	χ²=0.25 (df=1)	0.334

Table 14 identifies the individual clinical and laboratory variables significantly associated with mortality. Multi-organ failure showed the strongest association (26/35, 74.3% of non-survivors vs 4/40, 10.0% of survivors, χ²=29.52, p<0.0001), followed by an AARC score ≥10 (27/35, 77.1% vs 7/40, 17.5%, p<0.0001) and elevated serum lactate ≥2 mmol/L (25/35, 71.4% vs 8/40, 20.0%, p<0.0001). All twelve variables examined - spanning hepatic encephalopathy, oliguria, hyperbilirubinaemia, coagulopathy, renal dysfunction, hyponatraemia, hyperlactataemia, and elevated severity scores - were significantly more frequent among those who died.

**Table 14:** Clinical, laboratory, and severity-score predictors of in-hospital mortality *Chi-square test with Yates’ continuity correction (2×2 tables, df=1). INR: international normalized ratio; MELD: model for end-stage liver disease; SOFA: sequential organ failure assessment; AARC: ACLF Research Consortium.

Variable	Died (N=35)	Survived (N=40)	χ² (df=1)*	p-value
Hepatic encephalopathy present	25 (71.4%)	13 (32.5%)	9.81	0.0017
Oliguria / decreased urine output	24 (68.6%)	10 (25.0%)	12.6	0.0004
Total bilirubin >20 mg/dL	24 (68.6%)	12 (30.0%)	9.64	0.0019
INR >2.0	27 (77.1%)	13 (32.5%)	13.21	0.0003
Serum creatinine ≥2.0 mg/dL	19 (54.3%)	7 (17.5%)	9.59	0.002
Serum sodium <130 mEq/L	22 (62.9%)	9 (22.5%)	10.93	0.0009
Serum lactate ≥2 mmol/L	25 (71.4%)	8 (20.0%)	18	<0.0001
Serum lactate ≥4 mmol/L	12 (34.3%)	2 (5.0%)	8.7	0.0018
MELD ≥30	25 (71.4%)	14 (35.0%)	8.52	0.0035
SOFA ≥8	24 (68.6%)	8 (20.0%)	16.07	0.0001
AARC score ≥10	27 (77.1%)	7 (17.5%)	24.44	<0.0001
Multi-organ failure (≥2 extrahepatic OFs)	26 (74.3%)	4 (10.0%)	29.52	<0.0001

Table 15 examines complications and supportive-care requirements during hospitalisation. Hepatorenal syndrome (18/35, 51.4% vs 3/40, 7.5%, p<0.0001) and in-hospital sepsis (19/35, 54.3% vs 10/40, 25.0%, p=0.0182) were both significantly more frequent among non-survivors, whereas the need for invasive ventilation and renal replacement therapy did not differ significantly between the two groups (p=0.3808 and p=0.791, respectively), suggesting these interventions were used comparably in both groups without necessarily reflecting the eventual outcome.

**Table 15:** Complications and supportive-care requirements during hospital stay by in-hospital outcome *Chi-square test with Yates’ continuity correction (2×2 tables, df=1).

Variable	Died (N=35)	Survived (N=40)	χ² (df=1)*	p-value
Sepsis during hospital stay	19 (54.3%)	10 (25.0%)	5.57	0.0182
Hepatorenal syndrome	18 (51.4%)	3 (7.5%)	15.76	<0.0001
Need for invasive ventilation	8 (22.9%)	5 (12.5%)	0.77	0.3808
Need for renal replacement therapy	7 (20.0%)	6 (15.0%)	0.07	0.791

## Discussion

ACLF remains a dynamic and potentially reversible syndrome whose outcome is closely linked to the burden of organ failure as well as the severity of the underlying liver disease [1,3]. Despite advances in supportive critical care, it continues to carry high short-term mortality, particularly in resource-limited settings, and heterogeneity in diagnostic criteria and regional aetiological patterns has made institution- and region-specific data important for guiding clinical decision-making [2,4,5,7,8]. The present prospective observational cohort study characterises the clinical profile, precipitating factors, severity-score distribution, and factors associated with in-hospital mortality among 75 patients with APASL-defined ACLF at a tertiary care centre in India.

Demographic profile

ACLF predominantly affected males (66/75, 88.0%) in this cohort, with a mean age of 47.0 ± 13.7 years, consistent with the epidemiology of alcohol-related CLD in India. A comparable male preponderance has been reported from a large European cohort by Mikolasevic et al. (n=90), who attributed it to a higher prevalence of alcohol-related liver disease [15], and from Indian series: Behera et al. (n=120) reported a male-to-female ratio of 2.5:1 with a mean age of 44.9 ± 8.6 years [11], Halder et al. (n=132) reported 65.9% male patients with a median age of 40 years [17], and Arun et al. (n=51) reported 90.2% male patients with a mean age of 41.7 ± 6.8 years [14]. The consistency of male predominance and middle-age clustering across these studies, despite differing geography and diagnostic criteria, is in keeping with ACLF largely mirroring the epidemiology of alcohol-related chronic liver disease, and suggests it affects patients during their most economically productive years.

Clinical presentation

Jaundice (72/75, 96.0%) and ascites (68/75, 90.7%) were the commonest presenting features in this study, followed by hepatic encephalopathy (38/75, 50.7%) and oliguria (34/75, 45.3%). Behera et al. similarly reported jaundice in 100% and ascites in 88.3% of patients, with hepatic encephalopathy in 60% [11]. Arun et al. reported an almost identical pattern - jaundice in 100%, ascites in 98%, and encephalopathy in 56.9% of patients [14]. This concordance across geographically distinct Indian cohorts suggests that ACLF typically presents with advanced hepatic decompensation rather than an early or subtle clinical picture, underscoring the need for a high index of suspicion in any patient with known or occult CLD presenting with acute jaundice.

Aetiology and precipitating factors

Alcohol-related CLD was the leading aetiology in the present cohort (41/75, 54.7%), followed by HBV-related CLD (14/75, 18.7%). This mirrors the aetiological spectrum reported across Indian series: in a large cohort of 208 ACLF patients from Jaipur, Gawande et al. found alcohol to be the dominant underlying aetiology, with alcohol-related and hepatitis-related precipitants both contributing substantially to mortality [9], and Gupta et al. similarly reported alcohol as the leading cause of CLD among 100 ACLF patients graded by CANONIC criteria at a north Indian centre [10]. Continued alcohol consumption or superadded alcoholic hepatitis was the commonest acute hepatic insult in the present study (27/75, 36.0%), consistent with the pattern described by Gawande et al., while hepatitis E superinfection (12/75, 16.0%) and hepatitis B reactivation (12/75, 16.0%) reflect the continued endemicity of enterically and parenterally transmitted viral hepatitis on the Indian subcontinent. Infection (25/75, 33.3%), predominantly SBP, was the leading overall precipitating event in this cohort, a pattern echoed by El Sayed et al., who reported SBP as the dominant precipitant in 73.6-80.0% of ACLF patients admitted to an Egyptian intensive care unit [16], and by Halder et al., who identified sepsis (30.3%) as the commonest acute insult in an East Indian cohort [17]. Taken together, these findings are consistent with alcohol and bacterial infection - particularly SBP - being the two dominant, and potentially modifiable, precipitants of ACLF in this population; prospective interventional studies would be needed to confirm whether measures such as early diagnostic paracentesis or alcohol-cessation counselling alter outcomes.

Laboratory derangements and severity scores

Marked hepatic synthetic and excretory dysfunction (mean bilirubin 14.6 ± 7.2 mg/dL, INR 2.3 ± 0.8), thrombocytopenia, and renal and electrolyte derangement (mean creatinine 1.9 ± 1.2 mg/dL, sodium 131 ± 6 mEq/L) were evident at admission. The majority of patients had advanced disease at baseline, with 50/75 (66.7%) in CTP class C, 39/75 (52.0%) with a MELD score ≥30, and 34/75 (45.3%) with an AARC score ≥10. This pattern of late presentation with high baseline severity has been consistently reported: Choudhury et al., in the original AARC-score derivation and validation cohort of 1,402 patients, categorised ACLF into AARC grades I (5-7), II (8-10), and III (11-15), with 28-day cumulative mortality of 12.7%, 44.5%, and 85.9% respectively [6] - a gradient numerically similar to the mortality-by-grade relationship observed in the present cohort (14.3% [4/28], 51.9% [14/27], and 85.0% [17/20] in grades 1, 2, and 3; χ²=23.90, p<0.001), although, as noted in the Methods, ACLF grade in the present study was assigned by organ-failure count rather than by the AARC score band itself, so this comparison is illustrative rather than a direct replication.

Organ failure and outcome

Multi-organ failure (≥2 extrahepatic organ failures) was present in 30/75 (40.0%) of patients at baseline and, of the individual organ systems assessed by SOFA sub-score ≥3, cerebral (30/75, 40.0%) and coagulation (29/75, 38.7%) failure were the commonest extrahepatic failures. The present study demonstrated an in-hospital mortality of 35/75 (46.7%), with mortality rising steeply with ACLF grade and with the number of organ failures. Comparable short-term mortality figures have been reported from other Indian and regional cohorts: Halder et al. reported 28-day and 90-day mortality of 45.0% and 71.2% respectively [17]; Hareesh and Ramadoss reported 28-day and 90-day mortality of 45.0% and 52.5% in a south Indian cohort in which the CLIF-C ACLF score showed an area under the receiver operating characteristic curve (AUROC) of 0.86 for 28-day and 0.84 for 90-day mortality [13]; Kafle et al. reported an overall mortality of 56.9% in a Nepalese cohort in which oliguria, hepatic encephalopathy grade, ACLF grade, serum lactate, and the AARC score - but not the CTP score - were significantly associated with mortality on univariate analysis [19]; and El Sayed et al. reported ACLF grade-wise 1-month mortality of 20%, 60%, and 100% in grades 1, 2, and 3 respectively among ICU-admitted patients [16]. This convergence of findings across diverse settings is consistent with organ failure-based grading capturing clinically meaningful differences in the trajectory of ACLF, although, as none of these comparisons involved formal discrimination statistics (e.g. AUROC with confidence intervals) within the present cohort, the relative prognostic performance of organ failure-based versus liver-specific scores cannot be firmly established from this study alone.

Factors associated with mortality

On comparing survivors and non-survivors, the present study found significantly higher bilirubin, creatinine, INR, MELD, MELD-Na, SOFA, AARC score, and serum lactate, and significantly lower serum sodium, among non-survivors (all p ≤ 0.006), while age and sex showed no significant association with outcome on univariate analysis. Categorical analysis reinforced these findings: hepatic encephalopathy, oliguria, severe hyperbilirubinaemia, INR >2.0, renal dysfunction, hyponatraemia, elevated serum lactate, MELD ≥30, SOFA ≥8, AARC score ≥10, and multi-organ failure were all significantly more frequent among non-survivors, with multi-organ failure (26/35, 74.3% vs 4/40, 10.0%) and AARC score ≥10 (27/35, 77.1% vs 7/40, 17.5%) showing the strongest univariate associations (both p<0.0001). Because all analyses in this study were univariate, with no adjustment for confounding or for the overlap between individual laboratory variables and the composite scores that incorporate them, these findings should be interpreted as factors associated with mortality rather than as independent predictors. Comparable predictor profiles have nonetheless been reported from similar cohorts: Arun et al. found significantly higher MELD-Na, AARC, CLIF-OF, and CLIF-C ACLF scores among non-survivors and reported organ failure and higher MELD-Na and AARC scores to be associated with early death [14]; Hareesh and Ramadoss similarly found higher CLIF-C ACLF and CLIF-C organ failure scores, and a greater burden of coagulation failure, among non-survivors [13]; and Mikolasevic et al. reported elevated serum creatinine, systemic inflammatory markers, and multi-organ failure to be associated with mortality, with multi-organ failure carrying the strongest prognostic weight in their cohort (33 of 35 patients with multi-organ failure at admission died) [15]. Sepsis and hepatorenal syndrome, both significantly more frequent among non-survivors in the present cohort, have likewise been associated with mortality in ACLF in prior literature [1,3,15,17]; however, as noted in the Methods and in Table 15, these two variables, together with the need for invasive ventilation and renal replacement therapy, were ascertained during hospitalisation rather than at baseline, and may reflect consequences of clinical deterioration rather than baseline risk factors - their association with mortality in this dataset should therefore be interpreted with caution given the potential for reverse causation.

Clinical implications

These findings are consistent with a potential role for organ failure-based and dynamic severity scores - particularly the AARC and SOFA scores - alongside traditional liver-specific scores such as CTP and MELD in the risk stratification of patients with ACLF, in keeping with current AASLD practice guidance and the broader move towards harmonised, organ failure-inclusive definitions of ACLF [7,8]. However, because this study was observational, univariate, and lacked formal discrimination analysis, these findings should be regarded as hypothesis-generating; prospective studies with multivariable modelling and AUROC-based comparison of scores would be needed before firm recommendations on triage, referral for intensive care, or referral for liver transplantation could be drawn from this cohort alone.

Limitations

This study has several important limitations. It was a single-centre, observational cohort study with a relatively modest sample size, which may introduce selection bias and limit statistical power and generalisability of the findings to other settings. No patient underwent liver transplantation during the study period, so the relationship between severity scores and transplantation-free survival, or the performance of these scores in guiding transplant referral, could not be evaluated. All comparisons presented were univariate; no multivariable or multivariate regression analysis was performed, so the variables found to be statistically associated with mortality cannot be considered independent predictors, and the possibility of confounding by other, unmeasured or correlated variables cannot be excluded. Several of the laboratory variables analysed individually (e.g. bilirubin, creatinine, INR, serum lactate) are also components of the composite MELD, SOFA, and AARC scores analysed alongside them, introducing statistical overlap (collinearity) between these "independent" comparisons that was not formally accounted for. Complications and supportive-care variables such as sepsis, hepatorenal syndrome, invasive ventilation, and renal replacement therapy were ascertained during hospitalisation rather than at baseline and may represent consequences of disease progression rather than baseline predictors; their association with mortality is subject to potential reverse causation and should not be interpreted as evidence of a baseline prognostic role. Diagnosis and grading were performed using APASL/AARC criteria alone; parallel application of EASL-CLIF or North American Consortium for the Study of End-stage Liver Disease (NACSELD) criteria was not undertaken, which may account for some variation when comparing outcomes with cohorts using different diagnostic frameworks. Finally, long-term (90-day and beyond) outcomes and the discriminative accuracy (AUROC with confidence intervals) of individual severity scores were not formally assessed in this study.

## Conclusions

In this prospective observational cohort of patients with APASL-defined acute-on-chronic liver failure at a tertiary care centre in India, the syndrome predominantly affected middle-aged men and was most commonly attributable to alcohol-related chronic liver disease, with infection - chiefly SBP - and binge alcohol consumption as the leading precipitating events. Patients typically presented with advanced disease, reflected in high CTP, MELD, SOFA, and AARC scores at baseline. In-hospital mortality was high (35/75, 46.7%) and rose sharply with increasing ACLF grade. On univariate analysis, hyperbilirubinaemia, coagulopathy, renal dysfunction, hyponatraemia, hyperlactataemia, and elevated MELD, SOFA, and AARC scores were factors significantly associated with in-hospital death, with multi-organ failure and a high AARC score showing the strongest associations; sepsis and hepatorenal syndrome, ascertained during hospitalisation, were also associated with mortality but should be interpreted as potential markers of disease progression rather than baseline predictors. Because this was a single-centre study without multivariable analysis or liver transplantation, these findings should be regarded as hypothesis-generating. They support further, adequately powered, multivariable prospective studies to confirm whether organ failure-based prognostic scores can reliably identify high-risk patients with ACLF and inform timely escalation of care.
